# Accurate Characterization of the Adhesive Layer Thickness of Ceramic Bonding Structures Using Terahertz Time-Domain Spectroscopy

**DOI:** 10.3390/ma15196972

**Published:** 2022-10-07

**Authors:** Xiuwei Yang, Dehai Zhang, Biyuan Wu, Kaihua Zhang, Bing Yang, Zhongmin Wang, Xiaohu Wu

**Affiliations:** 1 Key Laboratory of Microwave Remote Sensing, National Space Science Center, Chinese Academy of Sciences, Beijing 100190, China; 2 University of Chinese Academy of Sciences, Beijing 100049, China; 3 Institute of Automation, Qilu University of Technology (Shandong Academy of Sciences), Jinan 250014, China; 4 Shandong Institute of Advanced Technology, Jinan 250100, China; 5 Basic Research Center, School of Power and Energy, Northwestern Polytechnical University, Xi’an 710072, China; 6 Henan Key Laboratory of Infrared Materials and Spectrum Measures and Applications, School of Physics, Henan Normal University, Xinxiang 453007, China; 7 Centre for Advanced Laser Manufacturing (CALM), School of Mechanical Engineering, Shandong University of Technology, Zibo 255000, China

**Keywords:** thickness measurement, terahertz time-domain spectroscopy, sparse deconvolution

## Abstract

Ceramic adhesive structures have been increasingly used in aerospace applications. However, the peaks of the signal on the upper and lower surface of the adhesive layer are difficult to measure directly due to the thin thickness of the adhesive layer and the effect of the attenuation dispersion of the ceramic layer. Thus, the existing non-destructive testing techniques have been ineffective in detecting adhesive quality. In this paper, the thickness of the adhesive layer is measured using terahertz time-domain spectroscopy. A sparse deconvolution method is proposed for the terahertz time-domain spectral signal of ceramic adhesive structures with different adhesive layer thicknesses. The results show that the methods proposed in this paper can realize the separation of reflection signals for glue layers with a thickness of 0.20 mm. By comparing with a wavelet denoising method and a modified covariance method (AR/MCM), the effectiveness of the sparse deconvolution method in estimating the thickness of the glue layer is demonstrated. This work will provide the theoretical and experimental basis for using terahertz time-domain spectroscopy to detect the homogeneity of ceramic adhesive structures.

## 1. Introduction

Novel ceramic materials have been widely used in the aerospace field. The flexible assembly of ceramic parts and metal structural parts has become a key technology to connect ceramic–metal components [1]. Glue layer connection technology is now widely used due to its advantages of uniform stress distribution, high connection efficiency, light weight, fatigue resistance and good sealing. The thickness and uniformity of the adhesive layer is essential for the strength of the connection between the components [2,3]. It is necessary to analyze the glue layer thickness uniformity for ensuring the functionality of the adhesive layer. In addition, it is important to save glue and reduce the contamination of the applied glue. The most crucial step in the evaluation of the glue layer uniformity is the acquisition of the exact thickness. Therefore, an accurate estimation of the intermediate glue layer thickness for ceramic–metal adhesive structures is of great importance.

Nondestructive testing techniques (NDT), which allow inspection without physical or chemical damage to the sample, have become increasingly important in recent years. At present, a variety of nondestructive testing techniques have been proposed to improve the measurement accuracy of thickness, such as ultrasonic [4] and X-ray [5] techniques. However, there are some limitations to each testing technology. These methods mentioned above mainly focus on the measurement of the thickness of the coating structures. Moreover, X-rays are potentially dangerous to humans during the testing process. Ultrasonic testing is only valid for thickness in sub-millimeter, which will have problems when measuring the thickness in micron units [6].

Terahertz time-domain spectroscopy (THz-TDS) is a new technique of spectroscopy measurement based on ultrashort pulse technology [7]. Compared to the traditional nondestructive testing techniques, THz-TDS can detect non-metallic materials and sub-micron structures [8]. More importantly, it is safe for the human body. Thus, THz-TDS is considered an alternative technique to avoid the limitations of traditional NDT methods [9].

The terahertz time-of-flight method is the key to the THz-TDS technique. When a light propagates through an interface between two media, reflections are generated [10]. In reflection mode, the exact time difference can be estimated by analyzing the arrival time of the short terahertz pulse signal to different interfaces of the sample [2,11]. Thus, the thickness of each layer can be calculated, making it the best means of detecting ceramic–metal glued structures.

However, there are two problems with this method. On the one hand, the echo signal may partially or completely overlap due to the thin thickness of the sample. On the other hand, the terahertz signal needs to penetrate the thick ceramic medium, which will scatter and attenuate the signal, making the signal-to-noise ratio of the echo signal decrease, both of which affect the accuracy of the adhesive layer thickness estimation [12].

To overcome these problems, various algorithms have been developed [13]. Recently, Chen et al. used the Wiener deconvolution method to process the terahertz echo signal to improve the signal time resolution, but the method is less effective for the thin layer [14]. Zhai et al. applied the autoregressive spectral extrapolation based on the modified covariance method for reconstructing the individual layer thicknesses, and the results are exciting [15]. However, this method requires a high signal-to-noise ratio in the signal region. When measuring the thickness of the rubber layer, it needs to pass through the thick ceramic layer, which has a great attenuation effect on the signal and a low signal-to-noise ratio, so it is difficult to select the signal segment with a high signal-to-noise ratio.

In this paper, a novel terahertz signal processing method based on sparse deconvolution is proposed to improve the detection accuracy of the glue layer thickness of ceramic parts. During the propagation of terahertz waves in the material, the ceramic material attenuates and disperses the terahertz waves, causing the terahertz pulse signal to spread and resulting in overlapping reflection peaks on the upper and lower surfaces of the adhesive layer, which has an impact on the estimation of time delays. To solve this problem, ceramic–glue–metal samples with three glue layer thicknesses are fabricated. The terahertz reflection signals are processed using wavelet denoising, AR/MCM and the sparse deconvolution method proposed in this paper, respectively. It was found that the estimated errors of the sparse deconvolution method are smaller and more effective and extracting the time delay is higher than the other two methods. This work has promising applications for improving the accuracy of the adhesive layer thickness of ceramic components in aerospace.

## 2. Theoretical Model

### 2.1. Transmission Mechanism of Terahertz Waves in Ceramic Glued Structures

**Figure 1:**
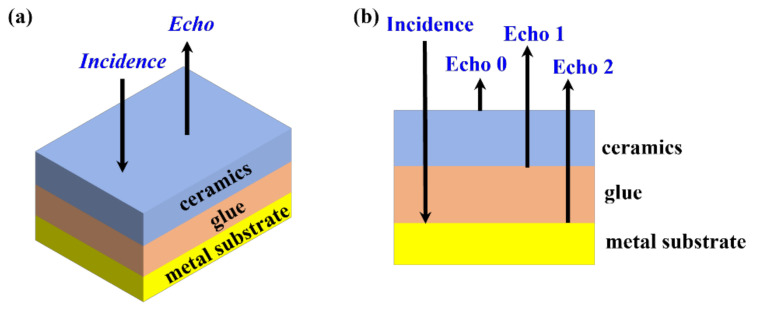
The model of the three-layered structure in this work: (**a**) 3D view; (**b**) the cross-sectional schematic diagram.

When terahertz waves propagate in different media, their transmission and reflection occur due to a change in the refractive index at the media interface [16]. The transmission of terahertz waves in a ceramic–glue structure is shown in Figure 1. Terahertz waves (Incident signal) are reflected on the ceramic surface to obtain Echo1 and then, penetrating the ceramic medium, are transmitted and reflected at the ceramic–glue layer interface (Echo 2) then transmitted terahertz waves are totally reflected at the lower surface of the glue layer (Echo 3), i.e., the glue layer–metal substrate interface. Terahertz reflection waves can be detected by a terahertz receiver [17]. The thickness of glue *d* for a single point can be described as:

(1) $$d=\frac{c}{2n}(T_{\mathrm{down}}-T_{\mathrm{up}})$$

where *n* is the refractive index of glue, *c* is the speed of light and T_down_ and T_up_ are the time-of-flight of terahertz waves on the upper and lower surfaces of the glue layer, respectively.

### 2.2. Principle of Sparse Deconvolution

Sparse deconvolution is used to obtain a more accurate terahertz time-of-flight in this work [18]. In the time domain, the THz reflected signal (electric field) *y*(*t*) is the convolution of the incident THz pulse *h*(*t*) with the impulse-response function *f*(*t*), which corresponds to the structure and properties of the sample at a given point of interest:

(2) $$y(t)=h(t)⊗f(t)=\int_{-\infty }^{+\infty }h(\tau )f(t-\tau )d\tau$$

For reflective THz imaging, the incident THz pulse *h*(*t*) can be obtained by first recording the THz signal reflected from a metal plate (THz reference signal) and then multiplying the reference signal by a factor of −1 for phase correction [19]. In practice, we should consider the discrete form of (2) with the sampling period *T_s_*:

(3) $$y_{n}=\sum_{m=0}^{M-1}h_{m}f_{n-m}+e_{n}$$

where $y_{n}=y(nT_{s})$, $h_{m}=h(mT_{s})$ and $e_{n}$ account for the noise originating from the measurement system and materials, with *n* and *m* as the indices of data points and *M* as the length of the data points. Let column vectors *y*, *h*, *f* and *e* collect the samples of $y_{n}$, $h_{n}$, $f_{n}$ and $e_{n}$, respectively [20]. Then, Equation (3) can be expressed as:

(4) y=Hf+e

where *H* is the convolution matrix whose rows are delayed versions of the reversed vector of $h^{T}$ or, equivalently, whose columns are delayed versions of *h*.

The basic idea of sparse deconvolution is to achieve the impulse response function by exploiting the sparse constraint. It aims at approximating the received THz signal *y* with *Hf*, where *f* is a sparse sequence, that is, *f* has only a few non-zero components. In this case, the sparse vector *f* can be computed by solving the $l_{0}$ regularized optimization problem, which is defined as:

(5) $$\underset{f}{\min}\frac{1}{2}{\left\|Hf-y\right\|}_{2}^{2}+\lambda {\left\|f\right\|}_{0}$$

where ${\left\|f\right\|}_{0}$ is the $l_{0}$-norm of f, which is defined to be the number of non-zero entries in f, and λ is the regularization parameter, which controls the trade-off between the sparsity of f and the residue norm.

Solving the non-convex $l_{0}$ regularized optimization problem, Equation (5) is known to be non-polynomial hard, and the global optimum cannot be guaranteed. It has already been shown [21] that this nonconvex optimization problem can be approximated with a convex optimization problem by replacing the $l_{0}$ penalty with the $l_{1}$ penalty as:

(6) $$\underset{f}{\min}\frac{1}{2}{\left\|Hf-y\right\|}_{2}^{2}+\lambda {\left\|f\right\|}_{1}$$

where ${\left\|f\right\|}_{1}$ is the $l_{1}$-norm of f, which is defined as the sum of the absolute values of its components. Since the $l_{1}$ norm is convex, a global optimum can be guaranteed.

The iterative shrinkage algorithm, which has been developed recently [22] and is able to address the above optimization problem effectively, is utilized in this paper. In General, in the iterative shrinkage algorithm, each iteration involves matrix–vector multiplication involving *H* and *H^T^*, followed by a shrinkage or soft-thresholding step. Specifically, the general iterative procedure is given by:

(7) $$f_{i+1}=S_{\lambda \tau }(f_{i}-\tau H^{T}(Hf_{i}-y))$$

where τ is an appropriate step size, which should obey:

(8) $$\tau <\frac{2}{{\left\|H^{T}H\right\|}_{2}}$$

In order to guarantee convergence, the shrinkage, or soft-threshholding, operator $S_{\lambda \tau }$ is defined as:

(9) $$S_{\lambda \tau }(f[n])=\left\{\begin{array}{ll} f[n]+\lambda \tau , & \;\;\;\;f[n]\leq -\lambda \tau \\ 0,\;\;\;\;\;\; & \;\;\;\;\left|f[n]\right|<\lambda \tau \\ f[n]-\lambda \tau , & \;\;\;\;f[n]\geq -\lambda \tau \end{array}\right.$$

A thorough theoretical analysis in [16] proves the convergence of this iterative shrinkage algorithm, guaranteeing that the solution is the global minimizer for convex *f*. Obviously, the time resolution of the obtained impulse response function *f* by sparse deconvolution depends upon the time resolution of the reference signal *h*, which is itself determined by the discretization precision corresponding to the data sampling period *T_s_*.

## 3. Experimental System and Samples

### 3.1. Experimental System

**Figure 2:**
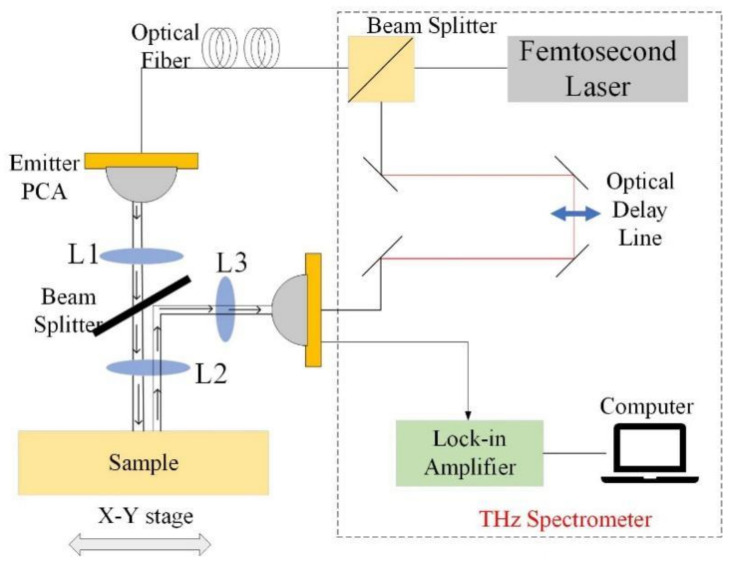
Instrument schematic diagram.

The reflection-mode operation of THz time-domain spectral (TDS) system (Quenda Terahertz Technologies, Qingdao, China) employed in the present study is illustrated schematically in Figure 2. The experimental device includes an ultra-fast femtosecond laser, an optical delay line, emitter and receiver photoconductive antennas (PCAs), a lock-in amplifier and a computer to control the device and process the signal. A 2 mm thick silicon wafer acts as a beam splitter to split the femtosecond laser into two beams, one for generating the terahertz wave and the other for sampling the terahertz wave. L1, L2 and L3 are, respectively, plano-convex polymethylpentene (TPX) lenses to collimate and focus the beam; PCAs to generate and receive THz radiation; and a beam to pump the emitter PCAs with a 780 nm femtosecond laser and probe the received THz wave with laser pulses split from the source and delayed by the optical delay line, by which time the sampled time signal has a step size of 0.01 ps and a time span of 120 ps. The PCAs are connected to the spectrometer by 780 nm single-mode optical fiber and the width of the THz pulse is approximately 1 ps. The effect of humidity on the system’s performance was limited by maintaining the relative humidity in the test environment at 25%, and the temperature was maintained at 25 °C [23,24].

### 3.2. Sample

**Figure 3:**
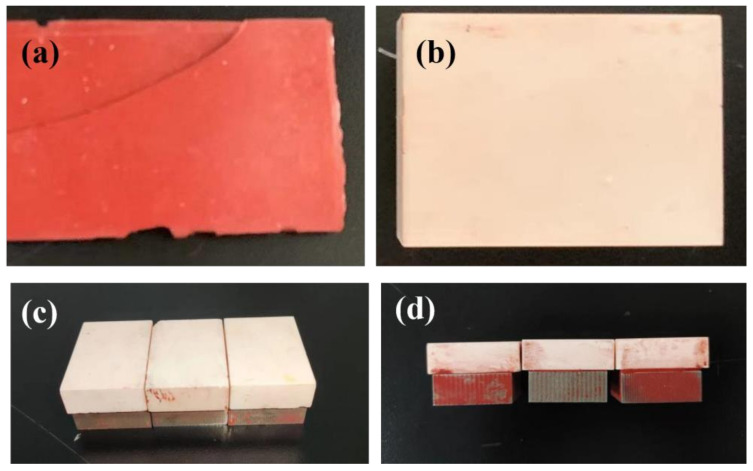
Physical map of samples: (**a**) glue; (**b**) ceramics; (**c**) real picture of sample with different adhesive thickness; (**d**) side view of the sample.

The structure used in the experiments consists of high-temperature-resistant quartz ceramics, a glue layer and metal substrates, shown in Figure 3. Figure 3a is a simple sample of an adhesive layer with a thickness of 2 mm, which is used to measure the refractive index of an adhesive layer. Figure 3b is a simple ceramic sample with a thickness of 10 mm, which is used for the measurement of the refractive index of the ceramic. Figure 3c,d are samples of ceramic and metal substrates bonded together by the adhesive process with a length and width of 60 mm × 20 mm. The sample bonding process and thickness control process are as follows: first the ceramic and metal substrates are cleaned with absolute ethanol, then they are dried with dry air to ensure that there are no impurities on the surface, and then the organosilicon rubber adhesive is prepared according to a certain proportion. After mixing evenly, the adhesive is coated evenly on the bonding surface between ceramic and metal with a special adhesive coating appliance (coating shovel). The coating thickness of the adhesive layer is controlled according to the bonding gap. Clearance control is carried out by presetting thick-limiting silk wire or precast adhesive strips of the same kind of adhesive with the required thickness on the contact surface. Then, the closed sample is tightened and fixed with a long tail clip or another special fixture.

Three kinds of samples with thicknesses of 1.00 mm, 0.50 mm and 0.20 mm were obtained. The samples were cured at a room temperature of 23 °C and an air humidity of 15% [25]. The reflective THz-TDS system was used to obtain the terahertz time-domain waveforms of the samples under test by reflective single-point inspection of each of the three glue layer thicknesses.

**Figure 4:**
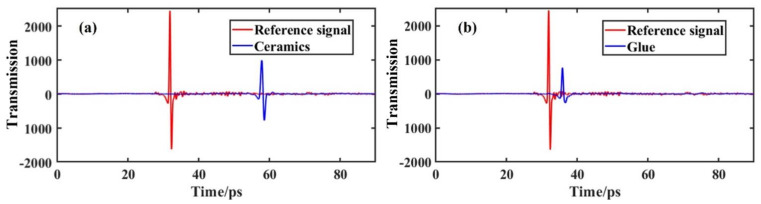
The transmission of (**a**) the ceramics and (**b**) the glue.

Figure 4a shows the transmission of the ceramics and the reference signal. Compared with the reference signal, one can see that the transmission peak of the ceramic is delayed in time and significantly decreased in amplitude, implying that the ceramic has an attenuating effect on the THz signal. The transmission of the glue is displayed in Figure 4b. A similar phenomenon can be seen in Figure 4a, though it is not described in detail here.

**Figure 5:**
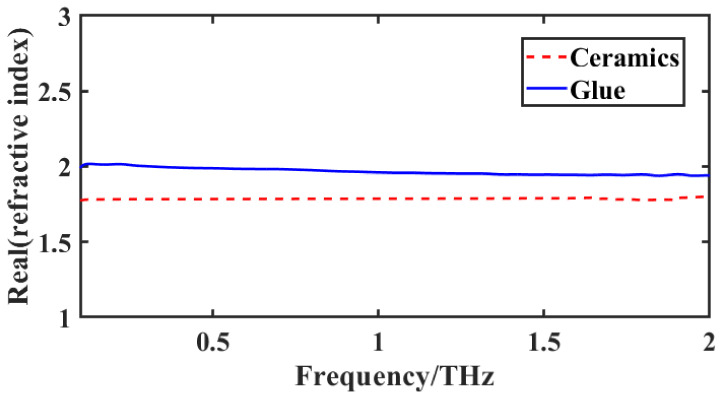
The real part of the refractive index for ceramics and glue.

According to the Ref. [26], transmission-mode terahertz detection is used to extract the optical parameters of the material. Figure 5 shows the real part of the refractive index for ceramics and glue. It can be found that the refractive index of the adhesive layer basically does not change with the frequency of the whole frequency segment, which can be regarded as a constant value. Therefore, the average refractive index of each frequency point is taken, and the average refractive index value is 1.98.

## 4. Results and Discussion

Here, the performance of the algorithm proposed is discussed with three samples of different thicknesses. The reflection data of three samples were obtained using the terahertz time-domain spectroscopy system described above.

**Figure 6:**
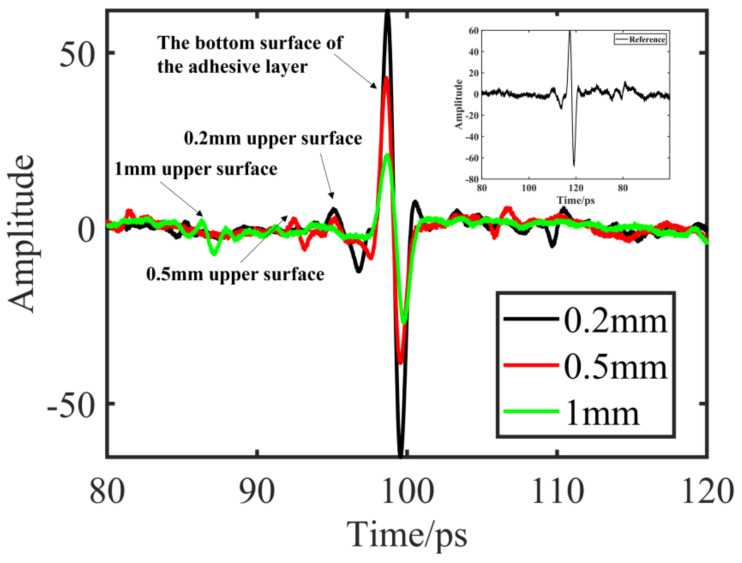
Terahertz time domain waveforms for three samples.

Figure 6 shows the THz time-domain waveforms of the three rubber layer thickness samples where the small window diagram is the reference signal obtained by the placed metal mirror. Each waveform is averaged 100 times at the same point of the sample to reduce the influence of random noise. The diagrams for each waveform signal of the two peaks, corresponding to rubber on the surface and under the surface, respectively, demonstrate that, because of the basal surface and metal bonding, the peak amplitude on the surface is larger, and on the surface with ceramic bonding, the peak amplitude is smaller; thus, it is more difficult to identify the peak position on the upper surface. In addition, it can be seen that the rubber thickness is 0.2 mm. The peak position of the upper surface is close to the peak position of the lower surface, which is more difficult to identify.

The reference signal is obtained by placing a metal plate at the location of the samples. Due to the noise effect, it is difficult to distinguish the echo signals of the upper and lower surfaces of the glue layer from original signal received from the experiment. Here, we use the wavelet denoising method (AR/MCM) and the sparse deconvolution method, proposed in this paper to process the original signal. For the wavelet denoising method, the sys8 wavelet base is applied [27]. After the original echo signal is pre-processed by wavelet transform, the AR/MCM is used to further process the data according to Refs. [28,29]. The part of the signal with high signal-to-noise ratio is selected and the optimal order p is determined.

**Figure 7:**
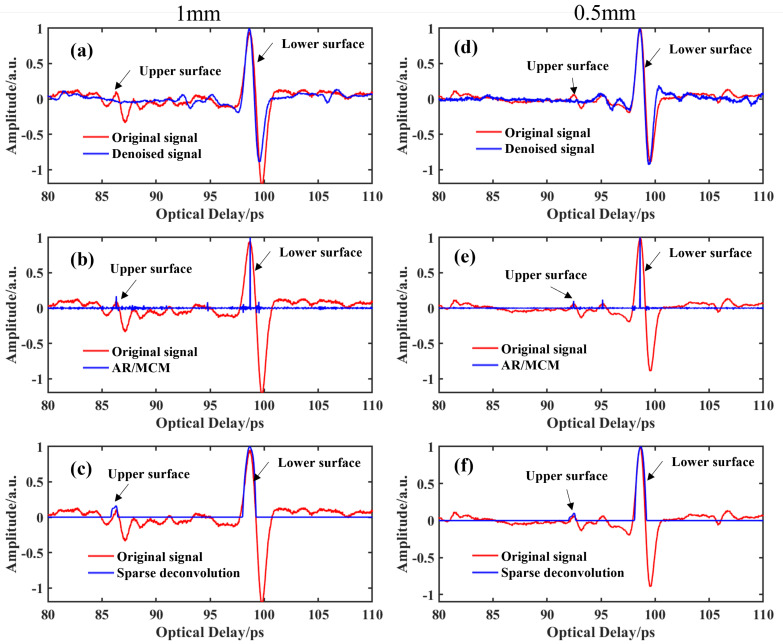
The results obtained by the different methods for the samples with different thicknesses of glue: (**a**–**c**) 1 mm; (**d**–**f**) 0.5 mm.

For the ceramic adhesive sample with 1.00 mm glue layer, Figure 7a–c show the signals processed by three different methods. Clearly, three methods can distinguish the two echoes corresponding to the upper and lower surfaces of the glue layer. Based on the time delay between the two echoes and the refractive index of the adhesive layer material in the terahertz band, the thickness of the glue layer can be estimated by Equation (1). The time delays between the time corresponding to the peak value of the upper surface of the adhesive layer and the time corresponding to the lower surface of the adhesive layer are (a) 12.57 ps, (b) 12.69 ps and (c) 12.95 ps for the three methods, respectively. The refractive index of the glue layer in the terahertz region is ~1.98. Furthermore, the calculated thicknesses of glue layers are (a) 0.95 mm, (b) 0.96 mm and (c) 0.98 mm, respectively. Because the actual thickness of the adhesive layer is 1 mm, the error between the actual thickness value and the value estimated by wavelet method, AR/MCM method and sparse deconvolution method is 0.05 mm, 0.04 mm and 0.02 mm, respectively. The thickness estimated by the sparse deconvolution method is closer to the actual value.

When the thickness of glue is 0.50 mm, the processed signal is shown in Figure 7d–f. Although all three methods can see the two echoes corresponding to the upper and lower surfaces of the gel layer, the envelope signal of the upper surface of the gel layer after the wavelet denoising method shows two peaks, which makes it more difficult to judge. In the subsequent signal processing, we take the highest peak value. According to Equation (1), the thickness of the glue layer can be estimated. The time delays between the first positive peak and the second positive peak are (a) 6.15 ps, (b) 6.31 ps and (c) 6.54 ps for the three methods. Thus, the calculated thicknesses of the glue layers can be calculated to be (a) 0.47 mm, (b) 0.48 mm and (c) 0.49 mm, respectively. The error between the actual thickness value and the value estimated by the wavelet method, AR/MCM method and sparse deconvolution method is 0.03 mm, 0.02 mm and 0.01 mm, respectively. The thickness estimated by the sparse deconvolution method is closer to the actual value.

**Figure 8:**
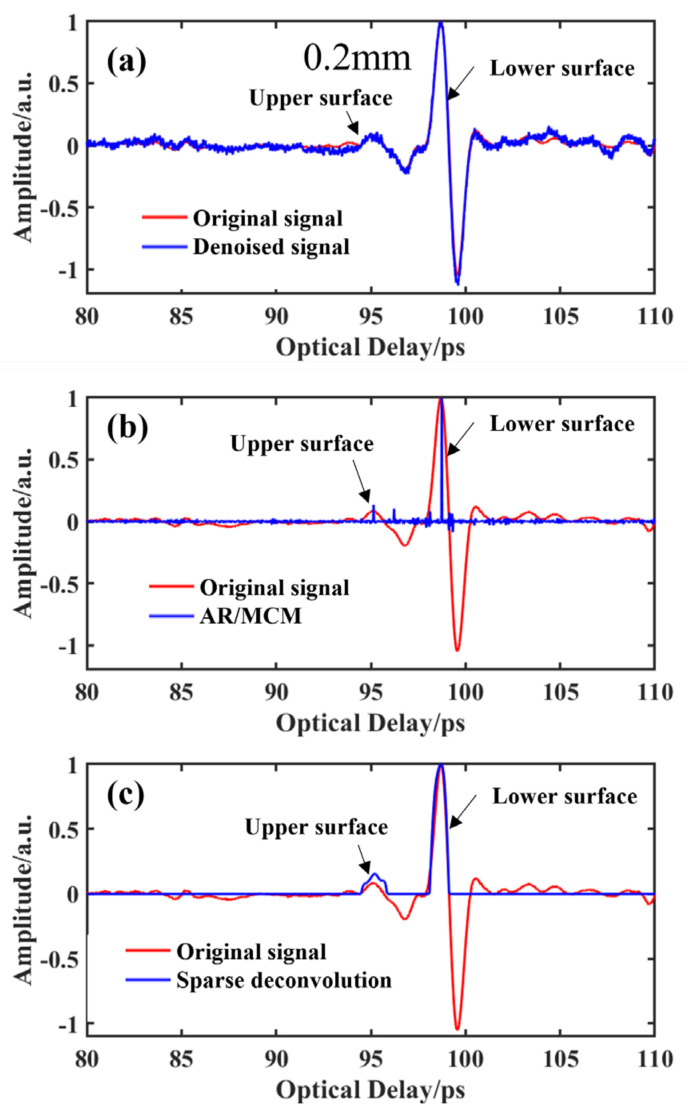
The results obtained by the different methods for the sample when the thickness of glue is 0.2 mm: (**a**) Wavelet method; (**b**) AR/MCM method; (**c**) Sparse deconvolution method.

As the thickness of the adhesive layer decreases, when the thickness of the adhesive layer is equal to 0.20 mm, the results for the three methods are exhibited in Figure 8a–c. It can be found that the wavelet denoising method cannot distinguish the peak of the envelope signal on the upper surface of the glue layer very clearly. However, two peaks at 95.59 ps and 96.66 ps can be obtained by the AR/MCM method, which will affect the accuracy of the later thickness calculation. The thickness of the glue layer can be estimated from Equation (1). For the three methods, the time delays between the first positive peak and the second positive peak are (a) 3.12 ps, (b) 3.1 ps and (c) 2.92 ps, respectively. The calculated thicknesses of the adhesive layer are (a) 0.24 mm, (b) 0.23 mm and (c) 0.22 mm, respectively. The error between the actual thickness value and the value estimated by the wavelet method, AR/MCM method and sparse deconvolution method is 0.04 mm, 0.03 mm and 0.02 mm, respectively. The thickness estimated by the sparse deconvolution method is closer to the actual value.

From Table 1, it can be concluded that the thickness of the adhesive layer calculated by the method proposed in this paper is closer to the actual thickness and the error is smaller. This error is mainly due to the influence of the ceramic layer and signal attenuation, resulting in a lower signal-to-noise ratio. Another reason is that the superposition of multiple reflections of the upper and lower interfaces leads to pulse broadening and error in determining the peak position. Terahertz signals are reflected several times within the ceramic layer and glue layer, causing an overlap of signals on the upper and lower surfaces of the glue layer. This phenomenon leads to the inability to accurately obtain the time delay between reflected signals at the interface, thus causing errors in the extraction and judgment of peaks.

## 5. Conclusions

In this paper, a sparse deconvolution method is proposed to solve the problem of thickness extraction of ceramic adhesive structures. Firstly, the principle of sparse deconvolution is analyzed, then the sample is prepared, the experiment carried out and the experimental results are processed and compared with the results of the wavelet denoising and AR/MCM methods. The wavelet denoising method makes the echo peak of the upper and lower interface of the rubber layer clear by reducing the noise, but the denoising effect of this method is limited. The AR/MCM method needs to select the appropriate frequency band range, and a too high or too low order will lead to the wrong peak, so there is a problem of poor stability. The sparse deconvolution method accurately reconstructed the impulse response function by using the selected regularization parameters and sparsity, and the reconstructed peak was sharp and without fluctuation. Compared with the other two methods, it extracted the adhesive layer thickness of ceramic adhesive structure more accurately, which proved that the method could effectively improve the practicability of THz nondestructive testing.

## Figures and Tables

**Table 1:** The comparison of calculated thickness from three methods.

The Real Value (mm)	Wavelet Method	AR/MCM	Sparse Deconvolution
1.0	0.95	0.96	**0.98**
0.50	0.47	0.48	**0.49**
0.20	0.24	0.23	**0.22**

## Data Availability

The data presented in this study are available on request from the corresponding author. The data are not publicly available because the research is still on going.
